# Copeptin: Limited Usefulness in Early Stroke Differentiation?

**DOI:** 10.1155/2015/768401

**Published:** 2015-06-08

**Authors:** Johannes von Recum, Julia Searle, Anna Slagman, Jörn Ole Vollert, Matthias Endres, Martin Möckel, Martin Ebinger

**Affiliations:** ^1^Division of Emergency Medicine, Department of Cardiology, Charité-Universitätsmedizin Berlin, 13353 Berlin, Germany; ^2^B.R.A.H.M.S GmbH, Thermo Scientific Clinical Diagnostics, 16761 Hennigsdorf, Germany; ^3^Department of Neurology, Charité-Universitätsmedizin Berlin, 10117 Berlin, Germany; ^4^Center for Stroke Research Berlin, Charité-Universitätsmedizin Berlin, 10117 Berlin, Germany; ^5^German Center for Neurodegenerative Diseases (DZNE), Charité-Universitätsmedizin Berlin, 10117 Berlin, Germany; ^6^German Centre for Cardiovascular Research (DZHK), Charité-Universitätsmedizin Berlin, 10117 Berlin, Germany

## Abstract

*Background*. Stroke can be a challenging diagnosis in an emergency-setting. We sought to determine whether copeptin may be a useful biomarker to differentiate between ischemic stroke (IS), transient ischemic attack (TIA), and stroke-mimics. *Methods*. In patients with suspected stroke arriving within 4.5 hours of symptom-onset, copeptin-levels were measured in initial blood-samples. The final diagnosis was adjudicated by vascular neurologists blinded to copeptin-values. *Results*. Of all 36 patients with available copeptin-values (median age 71 years, IQR: 54–76; 44% female), 20 patients (56%) were diagnosed with IS, no patient was diagnosed with hemorrhagic stroke, nine patients (25%) were diagnosed with TIA, and seven patients (19%) were stroke-mimics. Copeptin-levels (in pmol/L) tended to be higher in patients with IS [19.1 (11.2–48.5)] compared to TIA [9.4 (5.4–13.8)]. In stroke-mimics the range of values was extremely broad [33.3 (7.57–255.7)]. The diagnostic accuracy of copeptin for IS was 63% with a sensitivity of 80% and a positive predictive value of 64%. *Conclusion*. In this cohort of patients copeptin-levels within 4.5 hours of symptom onset were higher in patients with IS compared to TIA but the broad range of values in stroke-mimics limits diagnostic accuracy. This trial is registered with UTN: U1111-1119-7602.

## 1. Introduction

Thrombolysis and mechanical thrombectomy are currently the only proven effective treatments for patients in the early stage of acute ischemic stroke (IS) [1–4]. However, effects of both treatments are time-dependent. Therefore, fast diagnosis of IS is required. The clinical diagnosis of stroke in an emergency-setting can be challenging and prior to treatment imaging of the brain is mandatory. In most emergency departments (ED), only computed tomography (CT) is available to exclude hemorrhagic stroke. In these cases, the time-critical decision to start treatment is mainly based on the clinical impression of an acute stroke in the absence of intracerebral bleeds. Biomarkers may be helpful to differentiate between IS, TIA, and stroke-mimics.

One of the biomarkers that have recently gained attention is copeptin [5, 6]. Copeptin is a 39-amino-acid peptide and correlates with the secretion of Vasopressin (AVP). Not an organ-specific marker, copeptin increases early after acute hemodynamic stress [7, 8]. Copeptin-levels were shown to be elevated in patients with sepsis [9], acute respiratory diseases [10], and acute myocardial infarction [11].

In this exploratory pilot study, we analyzed acute copeptin-levels in patients who were admitted to the ED with suspected stroke. We sought to evaluate for the first time whether copeptin-levels differ between IS, TIA, and stroke-mimics.

## 2. Methods

The local ethics committee approved this study. Patients 18 years or older arriving within 4.5 hours of symptom-onset with suspected stroke as the cause of symptoms were eligible if code-stroke was activated. The code-stroke alarms the stroke unit, the CT staff, and the neurologist on duty in order to facilitate emergency treatment in stroke patients without contraindications. Routine blood-draw was performed at admission. Eligible patients were asked for written informed consent to participate in this study. Serum-samples from routine blood draw were then frozen at −80°C within 12 hours. Copeptin was measured in a single batch measurement using the B.R.A.H.M.S ultrasensitive (us) Copeptin KRYPTOR-assay after recruitment had finished. The assay time given by the manufacturer is 19 minutes. SPSS Statistics Version 20 was used for analysis. Patients' discharge-summaries were reviewed by a neurologist blinded to copeptin-levels to adjudicate the diagnoses IS, stroke-mimics, and TIA. The diagnosis of TIA followed the criteria of the World Health Organization defining TIA as rapidly developing clinical signs of focal or global disturbance of cerebral function, resolving within 24 hours in the absence of a nonvascular cause [12]. In case of visible lesions on brain imaging with resolving symptoms within 24 hours the term transient symptoms with infarction (TSI) was used [13].

## 3. Results

Between March and August, 2011, we enrolled 45 adult patients with an activated code-stroke in the ED. Of 36 patients (median age 71 years, IQR 54–76; 44% female) admission blood-samples were available for the current analysis. Of these, 20 patients (56%) were discharged with the final diagnosis of IS, 9 (25%) with TIA and 7 (19%) with stroke-mimics; no patient was diagnosed with hemorrhagic stroke or TSI. The final diagnoses in patients with stroke-mimics were epilepsy (*n* = 3), vestibular neuropathy (*n* = 1), migraine (*n* = 1), withdrawal delirium (*n* = 1), and loss of consciousness (not suggestive of TIA, MRI without evidence of stroke, *n* = 1). The median NIHSS-score of all patients was 4 (IQR 2–9). In patients with IS, the median NIHSS-score (7; IQR 3–11) was higher than in patients with stroke-mimics (6, IQR 1–9) or TIA (2, IQR 1–3). Mortality within one year was 11%. All patients who died (*n* = 4) were diagnosed with IS (copeptin-levels in pmol/L: 7.8/11.2/14.4/52.4, median of all survivors [*n* = 29]: 13.9). No patient died during the initial hospital stay. For patients' characteristics, see Table 1.

**Figure 1:**
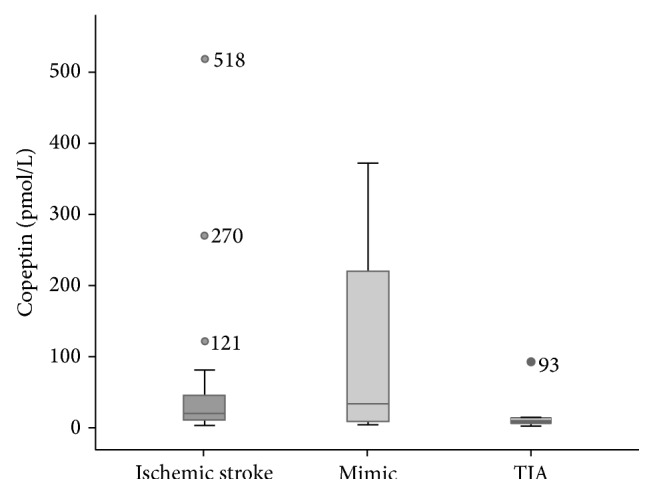
Copeptin-levels of patients with ischemic stroke, stroke-mimics, and TIA.

Median copeptin-level of all study patients on admission was 13.8 pmol/L (IQR 9.1–48.5). Median levels in patients with IS [19.1 pmol/L (IQR 11.2–48.5)] and in patients with stroke-mimics [33.31 pmol/L (IQR 7.57–255.7)] were higher than copeptin-levels in patients with TIA [9.4 pmol/L (IQR 5.4–13.8)] (*p* = 0.081; *p* = 0.153, resp., Figure 1). There was no significant correlation between copeptin values and NIHSS or age (NIHSS: Pearson *p* = 0.709, Spearman rho *p* = 0.076, age: Pearson *p* = 0.576, Spearman rho *p* = 0.907, resp.). Patients with stroke-mimics showed the largest range of copeptin-levels. Copeptin as a diagnostic marker at a predefined cut-off of 10 pmol/L yielded a sensitivity of 80% and a specificity of 44%. Using a cut-off of 14 pmol/L derived from previous cardiovascular studies [14, 15] sensitivity dropped to 55% and specificity increased to 69% (Table 2).

## 4. Discussion

This is the first analysis of very early copeptin-levels in patients with suspected stroke in an attempt to differentiate patients with IS from TIA and stroke-mimics. Previous stroke studies focused primarily on the prognostic value of copeptin [5, 16, 17]. All studies showed a correlation of copeptin and NIHSS score, lesion size, or ICH volume.

Patients with TIA showed the lowest copeptin-levels. Patients with stroke-mimics other than TIA had the highest copeptin-levels and displayed a wide range of values. Overall, this led to a low specificity for copeptin as a discriminatory marker for stroke at the cut-off level of 14 pmol/L. This cut-off level was derived from previous cardiovascular studies [14, 15] and coincides with the upper IQR boundary for patients with TIA in our cohort. The median copeptin-levels in a recently published stroke cohort (*n* = 783) within 12 hours from symptom-onset were 14.2 pmol/L (IQR 5.9–46.5). Although this copeptin value is close to the median copeptin value in our study, their IQR also shows a wide range. Stroke patients in this cohort with favorable outcome had a median copeptin-level below 10 pmol/L (9.6, IQR 4.7–25.8) [18]. A wide range of copeptin values in neurologic disorders has been observed before and is not clear yet. It may be influenced by different factors like individual levels of stress or the broad variety of neurologic disorders and needs to be further investigated. There is no specific clinical factor in our cohort causing the increase of the copeptin values in the mimic group. Sample calculations based on our findings revealed that more than 250 patients are required to detect significant differences between our three groups (power 80%). Given this relatively high number, the diagnostic usefulness of such a marker cannot be assessed appropriately with the data of this study and needs to be evaluated in further studies with more specific endpoints regarding lesion size and overall severity. The relatively short measurement time of 19 minutes qualifies copeptin as an excellent marker for the acute setting, if logistics at the institution allow for a short turnaround time.

## 5. Conclusion

In this cohort of patients with initially suspected stroke, copeptin-levels within 4.5 hours of symptom-onset were higher in patients with IS compared to TIA but the broad range of values in stroke-mimics limits diagnostic accuracy. Copeptin may aid prediction of stroke outcome but its usefulness in the differentiation between cerebral ischemia and stroke-mimics could not be proven positive in this small study. Further studies with larger sample sizes should be undertaken to elucidate whether the usefulness of copeptin in this setting adds diagnostic value to ease early decisions in the acute treatment of stroke.

## Figures and Tables

**Table 1:** Patients' characteristics.

Variables	All study patients (*N* = 36)	Patients with stroke (*N* = 20)	Patients with mimics (*N* = 7)	Patients with TIA (*N* = 9)
Female %	44	50	42.9	22

Age median (IQR)	71 (54/76)	68 (51/76)	59 (53/83)	71 (57/82)

Medical history:				

Previous stroke in % (*N*) (unknown = 1)	33 (12)	40 (8)	28 (2)	22 (2)

Previous TIA in % (*N*)	6 (2)	0	14 (1)	11 (1)

Copeptin in pmol/L Median (25%/75%)	13.8 (9.1/48.5)	19.1 (11.2/48.5)	33.31 (7.57/255.7)	9.4 (5.4/13.8)

tPA administered in % (*N*)	42 (15)	70 (14)	0	11 (1)

Stroke severity, median NIHSS score (25%/75%)	4 (2/9)	7 (3/11)	6 (1/9)	2 (1/3)

Length of inpatient stay in days Median (25%/75%)	8 (4/10)	9 (4/12)	6 (5/8)	6 (4/8)

TOAST score in % (*N*) Large artery atherosclerosis Cardioembolism Small vessel occlusion Other determined aetiology Undetermined aetiology Unknown	36.1 (13) 16.7 (6) 16.7 (6) — 2.8 (1) 27.8 (10)	55 (11) 15 (3) 15 (3) — — 15 (3)	— — — — — —	22.2 (2) 33.3 (3) 22.2 (2) — — 22.2 (2)

Risk factors in % (*N*) Hypertension Diabetes mellitus Hypercholesterolemia Neurologic disorders Coronary heart disease	83 (30) 33 (12) 19 (7) 22 (8) 11 (4)	80 (16) 35 (7) 20 (4) 15 (3) 20 (4)	86 (6) 29 (2) 14 (1) 43 (3) 0	67 (6) 33 (3) 22 (2) 22 (2) 0

Mortality within 1 year % (*N*) (unknown = 3)	11 (4)	19 (4)	0	0

**Table 2:** Ischemic stroke diagnostic performance of Copeptin at different cut-offs.

Patients with confirmed ischemic stroke						
	Number of patients above cut-off (total)	Sensitivity	Specificity	PPV	NPV	Accuracy
Copeptin 10 pmol/L	16 (20)	80%	44%	64%	63%	63%
Copeptin 14 pmol/L	11 (20)	55%	69%	69%	55%	61%
